# Characteristics of exercise capacity in female systemic lupus erythematosus associated pulmonary arterial hypertension patients

**DOI:** 10.1186/s12872-018-0783-7

**Published:** 2018-03-23

**Authors:** Bigyan Pudasaini, Guo-ling Yang, Chen Yang, Jian Guo, Ping Yuan, Yang Wen-lan, Rui Zhang, Lan Wang, Qin-Hua Zhao, Su-Gang Gong, Rong Jiang, Tian-Xiang Chen, Xiong Wei, Jin-Ming Liu

**Affiliations:** 1 Department of Pulmonary Function Test, Shanghai Pulmonary Hospital, Tongji University School of Medicine, Shanghai, 200433 China; 2 Department of Pulmonary Circulation, Shanghai Pulmonary Hospital, Tongji University School of Medicine, Shanghai, 200433 China; 3 Shanghai Pulmonary Hospital, Tongji University, School of Medicine, No. 507 Zhengmin Road, Shanghai, 200433 China

**Keywords:** Cardiopulmonary exercise testing, Oxygen uptake efficiency, PVR, Systemic lupus erythematosus associated pulmonary arterial hypertension, Idiopathic pulmonary arterial hypertension

## Abstract

**Background:**

To study the oxygen uptake efficiency and determine usefulness of submaximal parameters of oxygen uptake in systemic lupus erythematosus associated pulmonary arterial hypertension (SLE PAH) on performing a cardiopulmonary exercise test (CPET).

**Methods:**

CPET was performed in 21 SLE PAH patients, equal number of idiopathic pulmonary arterial hypertension (IPAH) patients and controls. Peak VO_2_, anaerobic threshold (AT), oxygen uptake efficiency slope (OUES) and oxygen uptake efficiency plateau (OUEP) and other CPET parameters were examined. All subjects had pulmonary function test (PFT) at rest, which included FEV_1_, FVC, FEV_1_/FVC, DLCO measurements. Right heart catheterization (RHC) was also done in SLE PAH and IPAH patients. CPET parameters were compared with RHC parameters to determine potential correlations.

**Results:**

Peak VO_2_, PETCO_2_ and peak O_2_ pulse were lower in SLE PAH than IPAH and controls with OUE being lower during all stages of exercise in SLE PAH. DLCO and FVC values were significantly lower in SLE PAH (*p* < 0.05). Peak O_2_ pulse and VO_2_@AT in SLE PAH and IPAH was low (*p* < 0.05) and significant difference between SLE PAH and IPAH was seen (*p* < 0.05). PVR correlated with the lowest V_E_/VCO_2_, O_2_ pulse, peak PETCO_2_ and OUE in SLE PAH patients (all *p* < 0.05).

**Conclusions:**

SLE PAH patients have cardiopulmonary exercise limitation with reduced oxygen uptake efficiency. VO_2_@ at AT, peak O_2_ pulse and O_2_ pulse at AT were significantly reduced (*p* < 0.05). Key CPET parameters correlated with elevated pulmonary vascular resistance (PVR). Submaximal parameters of oxygen uptake are equally useful in SLE PAH.

## Background

Systemic lupus erythematosus (SLE) is an autoimmune disease with heterogenous clinical manifestations. Pulmonary hypertension is a rarer complication of SLE, occurring in less than 5% of patients [1]. Almost half of SLE patients have some kind of pulmonary involvement and about a quarter of them have cardiac involvement during the course of their disease [2]. Pulmonary hypertension is an indicator of advanced disease in itself regardless of cardiac, pulmonary or of vascular origins. In a country like China with a humongous population even a low estimated incidence of pulmonary arterial hypertension (PAH) in SLE has a huge implication.

The ERS/ESC guidelines endorse the use of exercise testing for the diagnosis and treatment of pulmonary hypertension [3] and it has also been suggested that CPET variables have important clinical application for patients with PAH [4]. Although CPET can indirectly reflect gas exchange during a given exercise test, gas exchange and exercise capacity are closely coupled to both cellular and cardio-pulmonary mechanisms. This is central to all current and cited recommendations [5]. CPET’s ability to reflect quite accurately the VO_2_ and associated parameters [6] and its ability to provide reproducible assessment of functional capacity and treatment efficacy in patients [7] contributes to its growing clinical utility. CPET has been suggested for use both in diagnostic evaluation (Level B, Class II a), prognostication (Level B, Class II b), and determining therapeutic efficiency (Level C, Class II b) [6]. Use of oxygen uptake efficiency (OUE) in a submaximal exercise test has already been widely studied in patients with exercise limitation due to cardio-pulmonary diseases [8–11].

Despite numerous studies in combined connective tissue diseases, CPET data for both maximal and submaximal parameters for SLE PAH are still lacking. Thus, the primary objective of the present study was to study the OUE and determine usefulness of submaximal parameters of oxygen uptake in SLE PAH on performing a CPET and to correlate CPET measurements with hemodynamic variables as measured by the RHC.

## Methods

### Subjects

This study retrospectively enrolled 21 patients with SLE-PAH, 21 controls and an equal number of age, BMI and functional class matched IPAH patients at Shanghai Pulmonary Hospital from November 2009 to October 2015. Shanghai pulmonary hospital is a tertiary level specialty pulmonary hospital, thus confirmatory SLE diagnosis and its management was done at other centers, patients were on follow ups at those centers and stable at the time of evaluation. The diagnosis of pulmonary hypertension was based on clinical and laboratory data that included RHC to fulfill the currently accepted diagnostic criteria [12] (NICE, 2013). PAH was defined as a mean pulmonary arterial pressure (mPAP) ≥25 mmHg and a pulmonary arterial wedge pressure (PAWP) ≤ 15 mmHg [13] and with a normal or reduced cardiac output (CO), without known triggering factors. Subjects were excluded from the study if they had any evidence of: right-to-left cardiac shunt, history of coexisting lung diseases, forced vital capacity in the first second to the full vital capacity ratio (FEV_1_/FVC) < 65%, have undergone pulmonary endarterectomy or were anemic.

Patient demographics, their pulmonary function, N-terminal natriuretic peptide type-B (NT-pro BNP), haemodynamics and CPET data were collected.

### Cardiopulmonary exercise testing

CPET was performed on an electromagnetically braked cycle ergometer (VIASprint 150 P coupled to a Lab manager CPX cart, CareFusion, Jaeger crop, Hoechberg, Germany) using a breath-by-breath system to record gas exchange data over 10-s intervals. The protocol consisted of 3 min of rest, followed by 3 min of unloaded pedaling at 60 rpm, subsequently, a progressively increasing workload of 10 to 25 W/min to the maximum tolerance and finally 5 min of recovery. If patients developed fatigue, dyspnoea, chest tightness or any other discomfort during the CPET they were allowed to stop at anytime.

Measurements included workload, minute ventilation (V_E_), carbon dioxide output (VCO_2_), oxygen uptake (VO_2_), oxygen pulse (VO_2_/HR), heart rate (HR), heart rate reserve, breathing reserve (BR), respiratory exchange ratio (RER) and breathing frequency (BF). Anaerobic threshold (AT) which represents the beginning of anaerobic metabolism was determined by the V-slope method [14]. Minute ventilation/carbon dioxide output (V_E_/VCO_2_) slope was obtained by linear regression analysis of the relation between V_E_ and VCO_2_. OUES was computed using the following equation: VO2 = ‘a’ log VE + ‘b’, where ‘a’ = OUES [15]. OUEP was at 90 s of the highest consecutive values for VO_2_ (mL/min)/ V_E_ (L/min). Oxygen uptake efficiency at AT (OUE@ AT) was taken as the 60-s average of consecutive values at and immediately before the AT [16]. All predicted CPET values were calculated using accepted equations [17].

### Resting pulmonary function measurements

All subjects underwent resting measurements of forced vital capacity (FVC), forced expiratory volume in 1 s (FEV_1_), diffusing capacity of carbon monoxide (DLCO), forced expiratory flow at 25% of forced vital capacity (FEF_25_), forced expiratory flow at 50% of forced vital capacity (FEF_50_), and forced expiratory flow at 75% of forced vital capacity (FEF_75_) using ATS/ERS criteria with results reported in absolute terms and % predicted values (e.g., FVC% and PEF_50_%), residual volume (RV) and total lung capacity (TLC) using established standards [18–20] and equipment (Jaeger Corp., Hoechberg, Germany). All predicted spirometry values were calculated using standardized and accepted equations for Chinese [21].

### Right heart catheterization

All SLE PAH and IPAH patients underwent RHC with a true size thermodilution catheter (Edward 774) inserted via the left cubital fossa. Mean hemodynamic measurements included mPAP, PAWP, and mean right atrial pressure (mRAP). CO was obtained using the thermodilution method. Pulmonary vascular resistance (PVR) was calculated using standard formula: PVR = (mPAP-PAWP)/CO. Cardiac index (CI) was also calculated.

### Statistical analysis

Data were analyzed using SPSS 20.0 (SPSS Inc.; Chicago, IL). Continuous variables are presented as mean ± SD, and categorical variables are presented as a percentage. Most PFT and CPET values are expressed in absolute terms and %pred. Unpaired Student t test was used to identify differences between groups and to compare between the three groups at each time period one way analysis of variance (ANOVA) was used along with post hoc analysis. Correlations between CPET parameters and hemodynamic variables were determined by Pearson’s correlation test. *P* value of < 0.05 was considered significant.

## Results

### Demographics and pulmonary function test

Gender, age and body mass index (BMI) were similar between SLE-PAH, IPAH and control group (Table 1). The FVC and FEV_1_ were lower in SLE-PAH group as compared to IPAH and controls. FEV_1_/FVC was not much different between the groups, (SLE-PAH 83.7 ± 6.4, IPAH 81.1 ± 8.1 and control group 85.9 ± 6.6). The DLCO and its %pre in SLE-PAH and IPAH was not only significantly lower than in controls, there was a significance between the two patient groups as well [SLE PAH 12.7 ± 3.4, IPAH 17.1 ± 5.0, and control 19.2 ± 3.9, *p* < 0.05], indicating altered gas exchange efficiency. The decrease in TLC was not very remarkable between the groups; SLE-PAH patients exhibited more reduced values (SLE-PAH 3.8 ± 0.9, IPAH 4.64 ± 0.72, and controls 4.6 ± 0.5). Both the observed and %pred FVC was markedly reduced compared to the controls and a significant difference was noted between SLE PAH and IPAH as well (*p* < 0.05). (Table 1).

**Table 1:** Demographics, CPET and PFT parameters

	SLE PAH (*n* = 21)	IPAH (*n* = 21)	Control (*n* = 21)
Demographics			
Age, years	39.9 ± 9.6	36.7 ± 10.9	41.4 ± 12.8
Gender, F/M	21/0	21/0	21/0
Height, cm	157 ± 4.1	160 ± 7.2	159 ± 4.9
Weight, kg	53.8 ± 7.2	57.6 ± 9.2	57.7 ± 6.2
Body mass index(BMI), kg/m^2^	22.0 ± 2.9	21.1 ± 6.0	22.8 ± 2.6
CPET parameters			
Peak work load, W (%pred)	68.9 ± 22.9**	76.8 ± 23.1**	117.6 ± 21.1
	(66.3 ± 23.6)**	(54.0 ± 24.0)**	(108.4 ± 34.2)
Peak VO_2_,ml/min (%pred)	766 ± 258**	903 ± 207**	1335 ± 232
	(55.1 ± 18)**	(47.9 ± 22)**	(95.0 ± 40)
Peak VO_2,_ ml/min/kg	14.4 ± 4.9**	16.0 ± 3.5**	23.4 ± 4.3
Peak VCO_2_, ml/min	895.8 ± 333.1**	978.5 ± 269.8**	1549.1 ± 296.6
Peak HR, beats/min	147.3 ± 19.7	144.2 ± 26.1**	161.7 ± 18.1
Peak O_2_ pulse, ml/beat	5.2 ± 1.3**^#^	6.4 ± 1.6**^#^	8.2 ± 1.1
Peak VE, L/min	40.1 ± 10.8	46.4 ± 14.0	48.30 ± 11.8
Peak VO_2_/VE, ml/l	19.6 ± 4.6**	20.4 ± 5.1**	28.4 ± 4.8
Peak PETCO_2_, mmHg	26.9 ± 6.9**	25.46 ± 7.7**	37.5 ± 5.5
VO_2_AT, ml/min (%pred)	554 ± 182**^#^	678 ± 156**^#^	896 ± 171
	(53.9 ± 16.2)**	(57.3 ± 16.4)**	(85.9 ± 30.1)
AT O_2_ pulse, ml/beat	4.6 ± 1.1**	6.6 ± 3.9^#^	7.2 ± 1.4
Lowest VE/VCO_2_ (%pred)	44.7 ± 13.8**	43.56 ± 11.25**	29.74 ± 3.2
	(178.4 ± 57.1)**	(167.0 ± 42.6)**	(108.8 ± 13.4)
V_E_ /VCO_2_ Slope (%pred)	50.3 ± 28.9**	47.6 ± 17.4**	27.6 ± 4.3
	161.6 ± 92.6**	162.4 ± 71.6**	90.2 ± 16.1
OUES, L/min/log(L/min) (%pred)	0.96 ± 0.29**	0.97 ± 0.34**	1.76 ± 0.41
	(62.5 ± 19.2)**	(50.6 ± 26.5)**	(119.6 ± 61.8)
OUEP, ml/L (%pred)	26.2 ± 5.2**	27.2 ± 5.5**	37.5 ± 4.2
	(70.2 ± 12.8)**	(68.5 ± 14.2)**	(101.7 ± 13.3)
RER	0.89 ± 0.08	0.86 ± 0.07	0.84 ± 0.08
Pulmonary function test parameters			
Parameter	SLE PAH	IPAH	Control
FVC, L (%pred)	2.23 ± 0.6**^#^	2.80 ± 0.7*^#^	2.86 ± 0.5
	(72.1 ± 16.9)^**^	(79.0 ± 12.0)*	(92.5 ± 17.6)
FEV_1_, L (%pred)	1.86 ± 0.56**	2.28 ± 0.61*	2.44 ± 0.45
	(70.8 ± 17.3)*	(75.1 ± 12.0)**	(91.2 ± 11.3)
FEV_1_/FVC (%)	83.7 ± 6.4	81.1 ± 8.1	85.9 ± 6.6
MVV, L/min (%pred)	56.2 ± 20.6**	86.0 ± 25.1	77.9 ± 15.9
	(66.8 ± 23.6)**	(98.0 ± 19.0)*	(89.4 ± 18.1)
DL_CO_, ml/mmHg/min (%pred)	12.7 ± 3.4**^#^	17.1 ± 5.0^#^	19.2 ± 3.9
	(65.7 ± 16.7)**^#^	(85.2 ± 22.4^)#^	(90.9 ± 11.6)
TLC, L (%pred)	3.8 ± 0.9**^#^	4.6 ± 0.7^#^	4.6 ± 0.5
	(85.9 ± 14.4)*	(91.0 ± 10.0)	(96.9 ± 9.6)

*PFT* pulmonary function test, *FVC%* percent of predicted forced vital capacity, *FEV1%* percent of predicted forced expiratory volume in 1 s, *DLco%* percent of predicted gas transfer index or diffusing capacity for carbon monoxide, *MVV* minute ventilation volume, *TLC* total lung capacity, *CPET* cardiopulmonary exercise testing, *VO2* oxygen uptake per minute, *O2 pulse* oxygen pulse, *PETO2* partial pressure of end-tidal oxygen, *PETCO2* partial pressure of end-tidal carbon dioxide, *OUEP* oxygen uptake efficiency plateau, *OUES* oxygen uptake efficiency slope, *AT* anaerobic threshold, *RER* reserve expiratory ratio

*p < 0.05, **p < 0.005, between SLE PAH, IPAH Vs control; #*p* < 0.05 between SLE PAH and IPAH

### Cardiopulmonary exercise test measurements

Peak work load and the % pred peak work load in SLE-PAH were markedly reduced as compared to IPAH and controls. The peak VO_2_ and % pred peak VO_2_ in SLE-PAH was 766 ± 258 (55 ± 18), IPAH 903 ± 207 (47.95 ± 22.17) and control 1335 ± 232 (95 ± 40). The peak HR in SLE PAH was 147.3 ± 19.7, IPAH 144.2 ± 26.1 and control 161.7 ± 18.1. The VE/VCO_2_ slope in both patient groups in this study was significantly higher than in controls. The peak O_2_ pulse was also reduced in both patient groups, i.e. SLE PAH 5.2 ± 1.3, IPAH 6.4 ± 1.6, *p* < 0.05 VS 8.2 ± 1.1 in the controls and a statistically significant difference was seen between SLE PAH and IPAH as well (*p* < 0.05). The peak V_E_ was also reduced in both SLE PAH and IPAH, *p* < 0.05. Peak PeTCO_2_ were reduced in both SLE-PAH and IPAH. VO_2_ AT and its %pred was also markedly different in the three groups, i.e. SLE-PAH 554 ± 182 (53.9 ± 16.2), IPAH 678.2 ± 156 (57.3 ± 16.4) and control group 896 ± 171 (85.9 ± 30.1). Although the lowest V_E_/VCO_2_ and its %pred were almost comparable between SLE-PAH and IPAH and were significantly deranged when compared to the controls, it was nevertheless, correlated well with the PVR in both patient groups. The OUES and its %pred were also likewise deranged in SLE-PAH and IPAH as compared to the controls. The OUEP and %pred in SLE-PAH and IPAH was markedly reduced than in controls. The peak VO_2_ particularly correlated to OUES and OUEP in both SLE PAH and IPAH (*p* < 0.005). SLE PAH, OUES with peak VO_2_, r 0.78, *p* < 0.001, while in IPAH r − 0.62, *p* < 0.005 and SLE PAH, peak VO_2_ with OUEP, r 0.69, p 0.001, while in IPAH r 0.42, p 0.06). Significant correlation of OUEP % pred with peak VO_2_ was seen in SLE PAH group, i.e. SLE PAH r 0.68, p 0.001 and in IPAH r 0.24, p 0.276 respectively. OUEP % pred with peak VO_2_% pred in the SLE group was, r 0.54, p 0.01 while in IPAH was r 0.41, p 0.06. Ventilation at AT and V_E_/VCO_2_ slope were also significantly lower in SLE-PAH than in IPAH and controls (both *P* < 0.05, Table 1).

**Fig. 1:**
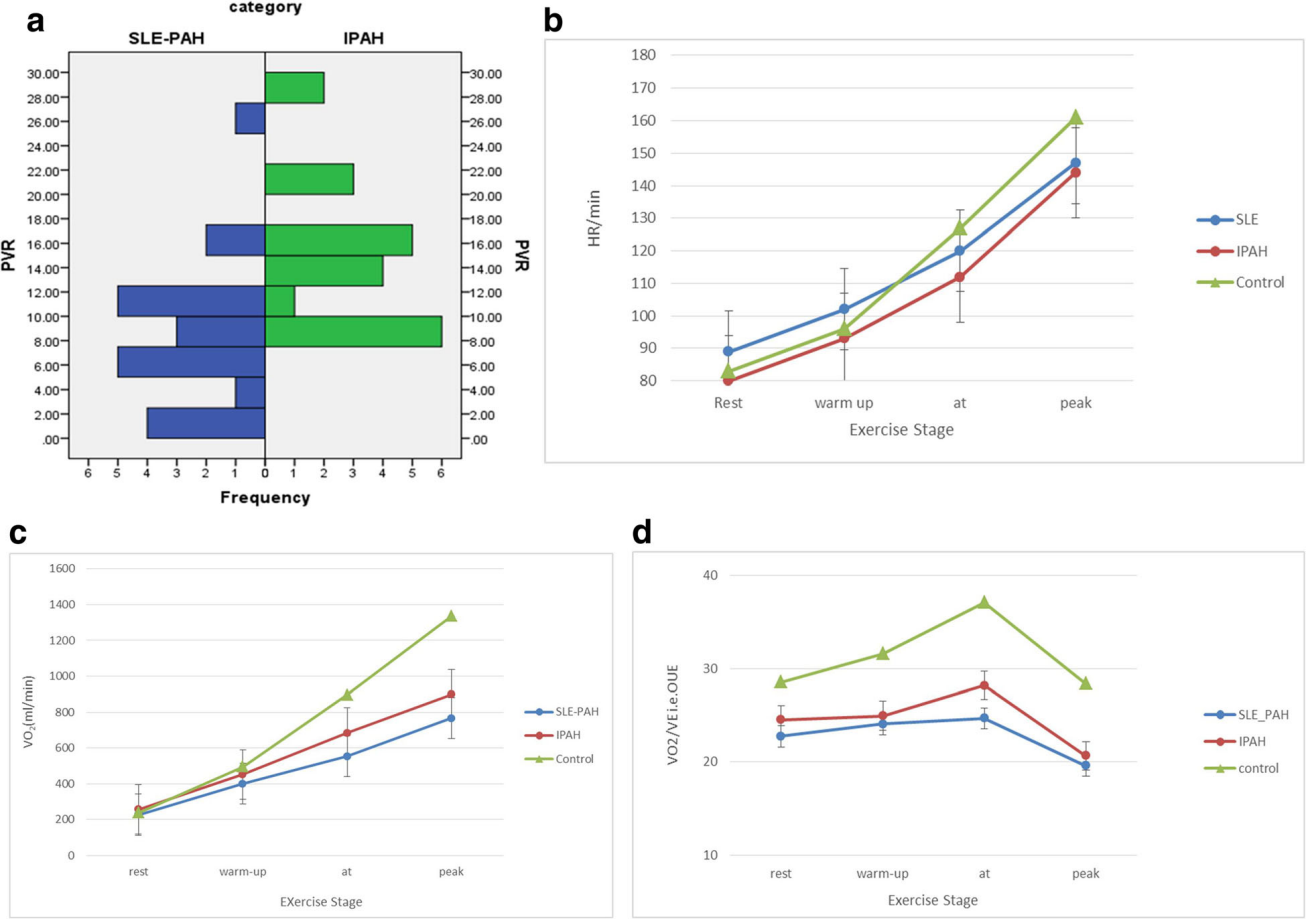
**a** PVR trends in SLE PAH and IPAH, **b** Heart rate during the different exercise stages in SLE PAH, IPAH & controls, **c** VO_2_ in SLE PAH, IPAH & controls, **d** VO_2_/V_E_ i.e. OUE in the three groups during different phases of CPET

Although HR increased as exercise intensity increased in all three cohorts the increase was not optimal in SLE-PAH and IPAH as compared to control subjects indicating an impaired chronotropic reserve in such patients (Fig. 1b). The peak PETCO_2_ was statistically significant between control and SLE PAH and IPAH and correlated with PVR in both patient groups. Correlations between key parameters are listed in Tables 3 and 4.

### Right heart catheterization

Right heart hemodynamics was compared between SLE-PAH and IPAH patients. The mPAP, RAP, PAWP and PVR were all lower in SLE PAH than in IPAH (*p* < 0.05). The CO in both groups was almost comparable. However, though the CI was lower in IPAH patients on an average it was not statistically significant (Table 2).

**Table 2:** Right Heart Catheterization (RHC) parameters

Parameter	SLE-PAH	IPAH	*p*
	(*n* = 21)	(*n* = 21)	
NYHA functional class (*n* = II/III)	2.4 ± 0.51 (12/9)	2.3 ± 0.48 (14/7)	0.5
NTproBNP, Pg/ml	1027 ± 1669	979 ± 1477	0.9
RAP, mm Hg	2.9 ± 2.4	5.1 ± 3.2	0.02
mPAP, mm Hg	41.3 ± 12.6	64.8 ± 14.1	< 0.001
PAWP, mm Hg	5.8 ± 3.4	7.7 ± 2.5	0.047
PVR, Wood unit	8.9 ± 5.8	14.1 ± 6.3	< 0.005
CO (L/min)	4.74 ± 1.4	4.73 ± 1.6	0.6
CI(Cardiac index),L/min/m^2^	3.07 ± 0.86	2.81 ± 0.69	0.24

*NYHA FC* New York Heart Association functional class, *NT-proBNP* n-terminal natriuretic peptide, *RHC* right-heart catheterization, *RAP* right atrial pressure, *mPAP* mean pulmonary arterial pressure, *PAWP* pulmonary artery wedge pressure, *CO* cardiac output, *CI* cardiac index, *PVR* pulmonary vascular resistance

On comparing the correlation of PVR with V_E_/VCO_2_ slope % pred and lowest V_E_/VCO_2_% pred in the two disease groups significant correlations were noted (Tables 3 and 4).

**Table 3:** Correlations between VE/VCO_2_ SLOPE (% pred) and Lowest V_E_/VCO_2_ (% pred) with some key parameters in SLE-PAH and IPAH patients

	VE/VCO2 SLOPE % pred		Lowest VE/VCO2% pred	
	SLE-PAH	IPAH	SLE PAH	IPAH
NT-proBNP, pg/ml	0.67**	−0.09	0.46*	0.10
NYHA	0.15	0.01	0.22	0.07
Peak vo2/kg	−0.49*	−0.12	− 0.54*	−0.42
Peak vo2%pred	−0.49*	−0.30	− 0.56**	− 0.61**
RAP, mm Hg	0.42	− 0.11	0.44*	− 0.03
mPAP, mm Hg	0.50*	0.45*	0.42	0.64**
CO, L/min	−0.61**	0.24	−0.67**	0.06
CI, L/min/m^2^	−0.65**	0.24	−0.73**	0.02
PVR, Wood U	0.76**	0.34	0.69*	0.62**
OUEP%pred	−0.80**	−0.62**	− 0.88**	−0.75**
OUES%pred	−0.61**	−0.31	− 0.64**	0.02

*NYHA* New York Heart Association heart failure classification, *mPAP* mean pulmonary artery pressure, *PVR* pulmonary vascular resistance, *CI* Cardiac index, *DL*_*CO*_ diffusing capacity for carbon monoxide; *Peak*$\overset{{}^{\circ}}{\mathsf{V}}$*O*_*2*_ peak oxygen uptake, *P*_*ET*_*CO*_*2*_ partial pressure of end-tidal carbon dioxide, *VE* ventilation, *VCO*_*2*_ carbon dioxide output

*p<0.05, **<0.005

**Table 4:** Correlation between PVR and key parameters in SLE PAH and IPAH

	PVR	PVR
	(SLE-PAH)	(IPAH)
Peak PETO2, mmHg	0.66**	0.54*
Peak PETCO2,mmHg	−0.63**	−0.47*
Peak WR, W	0.58**	− 0.21
Peak O2/HR, ml/beat	− 0.56**	− 0.40
Lowest VE/VCO2	0.70**	0.64**
Lowest VE/VCO2%	0.68**	0.62**
Slope%	0.76**	0.34
OUEP, ml/min	− 0.66**	−0.53*
OUEP%	− 0.65**	0.52
OUEP%pred	− 0.66**	−0.47*
OUES,L/min/log(L/min)	− 0.67**	0.54*
OUES%pred	− 0.46*	0.32

*P < 0.05; **P < 0.005

The NYHA functional class and CO were comparable between SLE PAH and IPAH but their mPAP, RAP, PVR and PAWP were different (Table 2, Fig. 1a). Although SLE PAH patients had lower mPAP, RAP, PVR and PAWP values there was no statistical difference with NTproBNP levels between SLE PAH and IPAH patients.

## Discussion

The clinical features of heart failure typically seen in the general population are less likely to be evident at presentation in patients with lupus [22]. PAH is the third leading cause of death in Chinese SLE patients [23] and the Chinese SLE treatment and research group (CSTAR) estimates the prevalence of PAH in SLE in china to be 3.8% with a very skewed female to male ratio (F:M = 10.1) [24]. The incidence was found to be 12% for CTD associated PAH and 25% for IPAH in our institution *(shanghai pulmonary hospital, unpublished data)*. Early identification and adequate treatment is particularly important as SLE-PAH patients by comparison have poorer prognosis [25]. It has also been demonstrated that PAH is an independent risk factor for mortality in SLE [26] and predisposes to increased cardiovascular events despite late onset or mild lupus [27]. CPET has proven very useful in noninvasively evaluating functional limitations in other patient groups. Thus, we sought to evaluate characteristics of CPET in SLE PAH.

Peak VO_2_ is reduced in patients with higher pulmonary vascular resistance and is highly correlated with the amount of functional pulmonary vascular bed [28]. When the cardiac compensation is not adequate both the peak VO_2_ and the O_2_ pulse are reduced, both of which were reduced in SLE PAH patients (Fig. 1c). After Baba et al. [15]proposed the usefulness of OUES, other parameters like OUEP %pred and lowest V_E_/VCO_2_% pred have since also been used as reliable indicators of exercise capacity in PAH patients [29–32] in addition to the peak VO_2_. We also noted peak VO_2_ to be particularly correlated to OUES and OUEP in both SLE PAH and IPAH (*p* < 0.005). OUE can be altered by CO, difference in pulmonary to systemic arterial oxygen tension, effectiveness of the respiratory membrane and the blood pH. The OUE is markedly impaired during all phases in SLE PAH patients and the peak is much more subtle than in IPAH or controls, which reflects the increased metabolic demand during the AT and peak not being met in such patients (Fig. 1d). This concurs with the report by Tan et al. [10] who reported similar results for IPAH patients. Despite cardiopulmonary reserve being adequate at rest, the OUE in SLE PAH is abnormal, resulting perhaps as a consequence of the increased pulmonary vascular resistance. We had reported similar findings in patients with pulmonary embolism [33].

Patients with a peakVO_2_ < 10 ml/kg per min are generally accepted to have a poor prognosis. A majority of our subjects had peak VO_2_ levels between 10 and 18 ml/kg per min. The V_E_/VCO_2_ slope in both the patient groups in this study was significantly higher than in controls. Corra et al. postulated that in such patients the V_E_/VCO_2_ slope may represent a proper descriptor of the heterogeneity of haemodynamics and neuro-humoral adaptations to exercise and disorders of ventilatory reflex control [34]. Based on their cut off values our patients in class II/III have to be classified as being at moderate risk for cardiac events.

SLE PAH patients had reduced FVC, FEV_1_, MVV and TLC. It has been suggested that a low %FVC also affects survival in PAH [35]. Though the extent of the disease could not be accurately quantified owing to lack of CT findings, based on the observed FVC in SLE PAH patients, suggests they have intermediate interstitial disease [36].

The peak VO_2_ was lower, the V_E_/VCO_2_ slope was higher and the PETCO_2_ was lower in SLE PAH patients, which is consistent with earlier reports, but what is not consistent is the magnitude of change in CPET variables was greater in SLE PAH rather than IPAH [6] in the current study. A reduced peak VO_2_, an increased V_E_/VCO_2_ slope with reduced ventilatory efficiency i.e. low peak/end exercise PETCO_2_ are clearly indicative of impaired pulmonary function and are key parameters of outcomes in PAH patients. Moreover peak PETCO_2_ has been reported to be an independent predictor of increased PVR in PAH patients [37] with similar findings being reported previously [38].

The increased pulmonary vascular resistance perhaps alters cardiac output (i.e. lower VO_2_@AT and lower peak O_2_ pulse) and pulmonary blood flow which in turn leads to reduced oxygen uptake as seen in our subjects, indicating that SLE PAH patients have markedly altered exercise capacity owing to cardiac and pulmonary factors.

The presence of pulmonary hypertension further enhances the decrease of DLCO indicating poor gas exchange even at rest [39–42]. IPAH patients with reduced DLCO were reported to have worse exercise performance despite having similar hemodynamic profiles [43]. This corroborates with our findings as well, furthermore the altered DLCO in SLE PAH was more severe in our patients. The reduced DLCO perhaps occurs as a combination of alterations in the respiratory membrane along with alteration in small pulmonary vessels [29]. It seems plausible that the reduced gas exchange efficiency in SLE PAH results more from pulmonary consequences as their mPAP, PVR and PAWP were lower than IPAH. PVR correlated better with other CPET parameters rather than RER in the present study (Tables 3 and 4), which is different from an earlier report where RER correlated better with PVR in IPAH [35]. Since the vascular resistance in SLE PAH is not as high as in IPAH, implying that SLE PAH patients perhaps develop more gradual cardiac consequences as compared to IPAH but faster and enhanced pulmonary/pulmonary vascular ones. As PVR is also related to lung volumes, being lowest at the functional residual capacity, the interstitial/restrictive pathology in the lungs due to SLE PAH in combination with small pulmonary vessel alterations perhaps contributes more towards the altered gas exchange efficiency. A lower PVR and better CI are an evidence of a bit better right heart hemodynamics in SLE PAH as compared to IPAH. Whether the slightly higher NTproBNP seen in SLE is a reflection of a difference in right atrial modification between the two disease groups or just an aberrant result needs to be explored further. Although beyond the scope of the current study it would be interesting to find out more about dynamic changes of the PVR during exertion in an optimally performed CPET.

Increased V_E_/VCO_2_ has been correlated with decreased cardiac output, elevated pulmonary arterial pressures, decreased alveolar capillary membrane conductance and diminished heart rate variability [43–45], we also noted correlations between PVR, V_E_/VCO_2_ SLOPE % pred and lowest V_E_/VCO_2_% in the two disease groups.

Though the hemodynamics data and NTproBNP levels were a bit higher than our subjects in earlier reports from China using CTD/SLE cohorts [46, 47], the demographics were comparable (Table 5). They used the 6MWD which is not adequate to judge activity levels and treatment response and thus we could not compare their results with ours for consistency as well. SLE PAH patients have distinct trends when performing a CPET. We agree with using established parameters like the peak VO_2_, PETCO_2_ and O_2_ pulse, but also recommend using the OUE (OUES, OUEP), as it is reliable when derived even from submaximal exercise.

**Table 5:** Review of demographics and methods in relevant recent studies

	2011 Jing [23]	2014 Zhang [46]	2014 Zeng [47]	2017 Liu^*current*^
RHC-dx SLE/CTD	35/103	62/129	111	21
Gender, F%	85.4	98.4	97.3	100
Age, yrs	41.5 ± 13.8	37.2 ± 12.2	34.63 ± 8.58	39.9 ± 9.6
mPAP, mm Hg	51.9 ± 15.6	49.6 ± 11.9	46.4 ± 11.4	41.3 ± 12.6
CI, L/min × m^2^	2.6 ± 0.9	2.8 ± 0.9	2.71 ± 0.78	3.07 ± 0.86
PVR, WU	12.9 ± 7.7	9.8 ± 7.2–12.2	10.5 ± 4.8	8.9 ± 5.8
6MWD,m	383.8 ± 106.7	394.7 ± 98.3	423.2 ± 92.4	–
NTpro BNP, pg/ml	–	(1171.4 ± 928.4)^a^	1767.9 ± 2128.4	1027 ± 1669
Peak work load, W (%pred)	–	–	–	68.9 ± 22.9
				(66.3 ± 23.6)
Peak vo2, ml/min (%pred)	–	–	–	766 ± 258
				(55 ± 18)
OUES, L/min/log(L/min)	–	–	–	0.96 ± 0.29
OUEP, ml/L	–	–	–	26.2 ± 5.2

^a^Overall all for CTDPAH

## Conclusions

In conclusion, SLE PAH patients have gas exchange impairment that is compounded by an inadequate cardiac compensation. This fact adds to the already known premise that SLE PAH patients have faster disease progression and worse prognosis. Since ours is a tertiary level referral center observer bias cannot be ruled out. This is a single center retrospective study with a small cohort of patients. The lack of imaging data limits our understanding regarding potential cardiovascular modifications and pulmonary processes occurring with the concurrent disease process. Although there is sufficient knowledge that incremental exercise tests in general are better than submaximal constant workload tests with regards to the intensity levels, we however note that in a study like ours employing a constant workload test would reflect more uniformly their exercise capacity. Despite this we have examined relevant parameters of CPET on a per kg body weight format where applicable to minimize bias. We also did not categorically calculate the SLE disease activity index or the medications they were already on at the time of the test. Nevertheless, we believe the results we have presented have future reference value. Further validations of these results are warranted to translate into clinical utility.

## Abbreviations

CI: Cardiac index
CO: Cardiac output
CPET: Cardiopulmonary exercise test
IPAH: Idiopathic pulmonary arterial hypertension
mPAP: Mean pulmonary artery pressure
mRAP: Mean right atrial pressure
NT-pro BNP: N-terminal natriuretic peptide type-B
OUE: Oxygen uptake efficiency
OUEP: Oxygen uptake efficiency plateau
OUES: Oxygen uptake efficiency slope
PAWP: Pulmonary arterial wedge pressure
Peak VO_2_: Peak oxygen uptake
PVR: Pulmonary vascular resistance
RER: Respiratory exchange ratio
RHC: Right heart catheterization
SLE PAH: Systemic lupus erythematosus associated pulmonary arterial hypertension
VCO_2_: Carbon dioxide output
V_E_: Minute ventilation
VO_2_: Oxygen uptake
VO_2_/HR: Oxygen pulse
